# Integrating Network Pharmacology and Metabolomics to Elucidate the Mechanism of Cryptotanshinone Against Platelet Aggregation

**DOI:** 10.3390/cimb47110953

**Published:** 2025-11-17

**Authors:** Jielan Huang, Zhenjie Liu, Baolin Wang, Haixin Qiu, Qiujie Chen, Jinyan Xian, Shen Liu, Xiaoxiu Shi, Ting Xia, Xiaoqing Tan, Wenhui Jiang, Yuanle Shen, Liuping Wang, Jianfang Feng

**Affiliations:** 1 School of Pharmacy, Guangxi University of Chinese Medicine, Nanning 530200, China; huangjielan3507@163.com (J.H.); rainman982@126.com (Z.L.); wbl15806025117@163.com (B.W.); qiuhaixin2020@163.com (H.Q.); 18376038045@163.com (Q.C.); xianjy2024@163.com (J.X.); 13878835241@163.com (S.L.); 18878634471@163.com (X.S.); xiating0226@163.com (T.X.); 18978305255@163.com (W.J.); shenyuanle2022@163.com (Y.S.); rousel@126.com (L.W.); 2 Guangxi Engineering Technology Research Center of Advantage Chinese Patent Drug and Ethnic Drug Development, Guangxi University of Chinese Medicine, Nanning 530200, China; 3 Guangxi Key Laboratory of Traditional Chinese Medicine Quality Standards, Guangxi Institute of Chinese Medicine & Pharmaceutical Science, Nanning 530200, China

**Keywords:** cryptotanshinone, platelet aggregation, network pharmacology, untargeted metabolomics, molecular docking, arachidonic acid metabolism

## Abstract

Cryptotanshinone (CTS), an antiplatelet compound from *Salvia miltiorrhiza*, exhibits in vitro potency comparable to aspirin. This study integrated network pharmacology and metabolomics to elucidate its underlying mechanisms. An acute blood stasis model was induced in Sprague-Dawley rats using epinephrine and ice-water immersion. Animals were assigned to seven groups. Platelet aggregation was measured turbidimetrically using arachidonic acid (AA) and adenosine diphosphate (ADP) as agonists. Core targets were predicted by network pharmacology, differential metabolites were screened, and pathways were enriched using untargeted metabolomics. Integrated analysis identified shared pathways and key targets, validated by molecular docking. AA- and ADP-induced aggregation was significantly increased in model rats versus the blank group. CTS at all doses markedly inhibited aggregation in a dose-dependent manner. Network pharmacology identified 15 core targets. Metabolomics identified 51 differential metabolites enriched in seven pathways, including glycerophospholipid and butanoate metabolism. Integrated analysis revealed five common pathways: linoleic acid metabolism, arginine biosynthesis, AA metabolism, glutathione metabolism, and drug metabolism—and four key targets (CYP3A4, NOS3, PTGS2, and GSTP1). Molecular docking showed strong binding energies (<−9 kcal/mol) between CTS and these targets. CTS inhibits platelet aggregation by regulating CYP3A4, NOS3, PTGS2, and GSTP1 and intervening in five metabolic pathways, supporting its potential as an anti-platelet agent.

## 1. Introduction

Platelet aggregation is the process by which activated platelets adhere via fibrinogen bridges, forming primary hemostatic plugs or pathological thrombi [1]. Dysregulation of this process drives various thrombotic events. Cardiovascular diseases (CVDs) remain the leading cause of mortality worldwide, with abnormal platelet activation and aggregation central to the pathogenesis of atherosclerosis, myocardial infarction, and ischemic stroke [2,3]. Antiplatelet agents are the cornerstones for preventing and treating these conditions, with aspirin and clopidogrel being the most widely used [4]. Despite their efficacy, both are associated with significant limitations: aspirin can induce dose-dependent gastrointestinal injury and bleeding, while clopidogrel resistance occurs due to CYP2C19 genetic polymorphism, P2Y12 receptor variations, or drug interactions, leading to reduced therapeutic efficacy and increased thrombotic risk [5,6].

In recent years, traditional Chinese medicine (TCM) has demonstrated a unique potential in antiplatelet therapy [7]. Its multi-component and multi-target mechanisms offer new insights into the development of antiplatelet agents [8]. Blood stasis, a central concept in TCM, is pathologically characterized by impaired circulation, blood stagnation, and hypercoagulability [9]. Therefore, identifying novel antiplatelet compounds from traditional medicinal plants with innovative mechanisms, high efficacy, and improved safety profiles has emerged as an important research direction and an urgent unmet need in cardiovascular pharmacology.

*Salvia miltiorrhiza Bunge*, a TCM herb with blood-activating and stasis-eliminating properties, is historically used to alleviate pain by unblocking meridians, clearing heart fires to relieve irritability, and cooling blood to reduce abscesses [10]. It has demonstrated remarkable clinical efficacy in cardiovascular disorders [11]. Cryptotanshinone (CTS), a liposoluble diterpenoid quinone and one of its primary active constituents, is considered a key bioactive substance responsible for these effects [12]. Recent studies have revealed that CTS exhibits broad pharmacological activities, including anti-inflammatory, antioxidant, antitumor, and cardiovascular-protective effects [13]. Accumulating evidence demonstrates that CTS strongly suppresses platelet aggregation, with in vitro activity comparable to that of aspirin, indicating its promising therapeutic potential for thrombotic disorders [14,15,16]. However, its antiplatelet mechanisms remain incompletely elucidated, thereby constraining its clinical development. Consequently, the molecular basis for CTS-mediated inhibition of platelet aggregation represents a focal point of ongoing investigation.

Elucidating the multi-component, multi-target, and multi-pathway actions of TCM poses a significant challenge, as conventional single-method approaches are often inadequate to fully capture the systemic pharmacological effects [17]. Network pharmacology addresses this challenge by constructing multidimensional “component-target-pathway” networks to predict core therapeutic targets and potential signaling pathways at the system level, providing hypotheses and directions for mechanistic studies [18,19]. Metabolomics complements this approach by providing a comprehensive analysis of global metabolic changes within an organism following drug administration, revealing altered pathways from the perspective of terminal metabolic responses [20]. The integrated application of network pharmacology and metabolomics involves a comprehensive pathway-level analysis of core targets and differential metabolites obtained from both omics approaches, achieving mutual validation and complementary enhancement [21].

This study aimed to systematically elucidate the mechanisms by which CTS inhibits platelet aggregation using an integrated approach combining network pharmacology and metabolomics. Specifically, network pharmacology was employed to predict core targets and pathways, whereas untargeted metabolomics profiled metabolic alterations in platelets following CTS treatment, identifying significantly dysregulated metabolites and their associated metabolic pathways. Integrating the results from both omics analyses enables identification of the key targets and metabolic pathways involved in the antiplatelet aggregation effect of CTS. Furthermore, molecular docking was conducted to simulate the binding mode between CTS and key targets identified through integrated analysis, evaluating the binding affinity and stability at the atomic level. This computational validation provides structural insights for future experimental validation.

## 2. Materials and Methods

### 2.1. Drugs and Reagents

Aspirin enteric-coated tablets (positive control, batch No. BJ60885) were purchased from Bayer Healthcare Co., Ltd. (Beijing, China). Clopidogrel bisulfate tablets (positive control, batch No. AA984) were obtained from Sanofi (Hangzhou) Pharmaceutical Co., Ltd. (Hangzhou, China). Cryptotanshinone (purity ≥ 98%, batch No. PS010099) was supplied by Chengdu Pusi Biotechnology Co., Ltd. (Chengdu, China). Epinephrine hydrochloride injection (batch No. 62111202) was acquired from Suicheng Pharmaceutical Co., Ltd. (Xinxiang, China). Physiological sodium chloride solution (batch No. L220091206) was provided by Sichuan Kelun Pharmaceutical Co., Ltd. (Chengdu, China). Sodium carboxymethyl cellulose (CMC-Na, batch No. 20191222) was purchased from Tianjin Bodi Chemical Co., Ltd. (Tianjin, China). Adenosine diphosphate (ADP, batch No. 3215364) and arachidonic acid (AA, batch No. 3215335) were procured from Beijing Baibin Medical Device Co., Ltd. (Beijing, China). LC-MS grade acetonitrile and methanol were obtained from CNW Technologies (Shanghai, China). LC-MS grade ammonium acetate was supplied by Sigma-Aldrich (St. Louis, MO, USA). LC-MS grade ammonia solution was purchased from Fisher Chemical (Shanghai, China). Ultrapure water was provided by Guangzhou Watsons Food & Beverage Co., Ltd. (Guangzhou, China).

### 2.2. Animal Experimentation

#### 2.2.1. Experimental Animals and Grouping

Seventy male Sprague–Dawley rats (250 ± 10 g) were obtained from Beijing Vital River Laboratory Animal Technology Co., Ltd. (Beijing, China; license No. SCXK2021-0011). Animals were maintained in a specific pathogen-free (SPF) barrier facility (20–25 °C, 40–60% relative humidity, 12 h light/dark cycle) with ad libitum access to standard chow and autoclaved water. All procedures were approved by the Guangxi University of Chinese Medicine Laboratory Animal Ethics Committee (Approval No: SYXK-2019-0001; Approval date: 1 November 2019) and conducted in accordance with the approved protocol and institutional animal-welfare guidelines.

After a 7-day acclimatization period, rats were randomly assigned to seven groups (*n* = 10 each). Mortality during the experiment reduced the final numbers to: (1) control (C) (*n* = 10), (2) model (M) (*n* = 8), (3) clopidogrel (LB, 30 mg kg^−1^) (*n* = 7), (4) aspirin (AS, 100 mg kg^−1^) (*n* = 7), and (5) low-dose CTS (Y05, 0.5 mg kg^−1^) (*n* = 10); (6) medium-dose CTS (Y5, 5 mg kg^−1^) (*n* = 6); and (7) high-dose CTS (Y50, 50 mg kg^−1^) (*n* = 10).

#### 2.2.2. Drug Administration and Model Establishment

Rats in the drug-treated groups received the respective agents by intragastric gavage once daily for five consecutive days. The C and M groups received an equal volume of 0.5% (*w*/*v*) sodium carboxymethylcellulose. On day 5, 1 h after the final gavage, all groups except C were intraperitoneally (i.p.) injected with adrenaline hydrochloride (1 mg mL^−1^, 0.8 mL kg^−1^); group C received an equal volume of 0.9% saline. Two hours later; the rats in the model and drug-treated groups were immersed in ice water (0–4 °C) for 4 min; with their heads kept above the water and then dried thoroughly. After an additional 2 h; a second intraperitoneal injection of adrenaline hydrochloride (or saline for group C) was administered to induce acute blood stasis [22]. All the rats were fasted for 12 h with free access to water. Successful modeling was confirmed by the appearance of typical signs: dark purple claws and tails; pale ears; and cold aversion

#### 2.2.3. Sample Collection and Processing

After a 12 h fast, the rats were anesthetized by the intraperitoneal injection of 20% urethane at 1 g/kg, and blood was collected from the abdominal aorta. Samples for metabolomics were collected into plain vacuum tubes, and samples for platelet aggregation assays were collected into sodium citrate anticoagulant tubes.

For metabolomics, blood in plain tubes was incubated at room temperature for 1 h, centrifuged at 3000 rpm for 10 min, and the supernatant was transferred to sterile 1.5 mL EP tubes. This was further centrifuged at 12,000 rpm at 4 °C for 10 min, and the resulting supernatant was aliquoted into sterile 1.5 mL tubes and stored at −80 °C until further analysis.

### 2.3. Platelet Aggregation Assay

Platelet aggregation was measured using light-transmission aggregometry [23]. Platelet-rich plasma (PRP) and platelet-poor plasma (PPP) were prepared as follows: whole blood was left at room temperature and centrifuged at 600 rpm for 10 min; this step was repeated twice, and the supernatant was collected into 1.5 mL EP tubes as PRP. The remaining plasma was centrifuged at 3000 rpm for 15 min at room temperature, and the resulting supernatant was collected into 1.5 mL EP tubes as PPP.

The aggregometer was pre-warmed to 37 °C for 15 min. After blanking with 200 μL PPP, 200 μL PRP was placed in a cuvette and incubated at 37 °C for 5 min. Subsequently, 25 μL ADP and 25 μL AA were added as agonists, followed by a magnetic stir bar. The maximum aggregation rate of PRP was recorded within 5 min following agonist addition at 37 °C.

### 2.4. Network Pharmacology

#### 2.4.1. Collection of Potential Compound Targets

Using “cryptotanshinone” as the keyword, the 2-D structure, SMILES string, and InChIKey were retrieved from PubChem (https://pubchem.ncbi.nlm.nih.gov/, accessed on 9 October 2024). Putative human targets were predicted using the Comparative Toxicogenomics Database (CTD) (https://ctdbase.org/, accessed on 9 October 2024) and TCMSP (https://www.tcmsp-e.com/, accessed on 10 October 2024) and Swiss Target Prediction (http://swisstargetprediction.ch/, accessed on 10 October 2024), with the species restricted to Homo sapiens.

#### 2.4.2. Disease Target Acquisition

Targets associated with the keyword “anti-platelet aggregation” were extracted from OMIM (http://www.omim.org/, accessed on 10 October 2024), STRING (http://string-db.org/, accessed on 10 October 2024), GeneCards (https://www.genecards.org/, accessed on 10 October 2024), and DrugBank (https://go.drugbank.com/, accessed on 10 October 2024), and converted using UniProt (https://www.uniprot.org/, accessed on 10 October 2024). The drug- and disease-related targets were intersected using Venny 2.1.0 (https://bioinfogp.cnb.csic.es/tools/venny/index.html, accessed 10 October 2024) to generate common targets, which were visualized in Venn diagrams.

#### 2.4.3. Protein–Protein Interaction Network Construction and Core Target Selection

Intersected targets were uploaded to STRING (http://string-db.org/, accessed 11 October 2024) with species set to Homo sapiens and a confidence threshold of 0.4 to generate the PPI network. The resulting data were imported into Cytoscape 3.10.0 software for visualization. Node size and color denote degree values, while edge width and color represent combined scores.

#### 2.4.4. GO and KEGG Enrichment Analysis

Intersected targets were analyzed using DAVID (https://davidbioinformatics.nih.gov/, accessed 11 October 2024) for Gene Ontology (GO) and Kyoto Encyclopedia of Genes and Genomes (KEGG) enrichment, with Homo sapiens as background. GO terms were ranked by *p*-values, and the top ten biological processes were plotted as bar charts. Likewise, KEGG pathways were ranked by *p*-value, and the top 30 were visualized in Sankey diagrams.

### 2.5. Untargeted Metabolomics Analysis

#### 2.5.1. Extraction of Serum Metabolites

Frozen serum aliquots stored at −80 °C were thawed on ice and allowed to equilibrate to room temperature. A 100 μL volume of each sample was transferred to a 1.5 mL EP tube, followed by the addition of 400 μL ice-cold extraction solvent (methanol/acetonitrile 1:1, *v*/*v*) containing a cocktail of stable-isotope-labeled internal standards. The mixture was vortexed for 30 s and then sonicated in an ice-water bath for 10 min. After incubation at −40 °C for 1 h, proteins were pelleted by centrifugation at 12,000 rpm (13,800× *g*, 8.6 cm rotor radius) for 15 min at 4 °C. The resulting supernatant was transferred to an LC–MS autosampler vial for analysis. An equal volume of the supernatant from each sample was pooled to generate a pooled quality control (QC) sample, which was analyzed alongside the study samples throughout the batch.

#### 2.5.2. LC-MS Analysis Conditions

Chromatography was performed on a Vanquish UHPLC system (Thermo Fisher Scientific, Waltham, MA, USA) equipped with a Waters ACQUITY UPLC BEH Amide column (2.1 mm × 100 mm, 1.7 µm). Mobile phase A consisted of 25 mmol L^−1^ ammonium acetate and 25 mmol L^−1^ aqueous ammonia in water; mobile phase B was acetonitrile. The column temperature was maintained at 30 °C, the autosampler tray was maintained at 4 °C, and the flow rate was set at 0.5 mL min^−1^. The injection volume was 2 L. Gradient elution was as follows: 0–0.5 min, % B; 0.5–7 min, 95–65% B; 7–8 min, 65–40% B; 8–9 min, 40% B; 9–9.1 min, 40–95% B; 9.1–12 min, 95% B.

Mass spectrometry was performed using an Orbitrap Exploris 120 mass spectrometer (Thermo Fisher Scientific) controlled by Xcalibur software (v. 4.4, Thermo Fisher Scientific, Waltham, MA, USA). Full-scan and tandem mass spectra were acquired in the positive-ion and negative-ion modes. Key parameters were: sheath gas flow rate (50 arbitrary units); auxiliary gas flow rate (15 arbitrary units); capillary temperature 320 °C; full-MS resolution, 60,000; MS/MS resolution, 15,000; normalized collision energies, 10, 30, and 60%; spray voltage, 3.8 kV (positive) or −3.4 kV (negative).

#### 2.5.3. Data Processing and Analysis

Raw data were converted to mzXML format using ProteoWizard software (v. 3.0.21120; https://proteowizard.sourceforge.io, accessed on 8 November 2024) and subsequently processed with the XCMS-based R package (v. 4.1.2; R Foundation for Statistical Computing, Vienna, Austria) for peak detection, extraction, alignment, and integration. Metabolites were annotated by matching the MS^2^ spectra against the BiotreeDB (version 2.1) database, using an algorithm score cutoff of 0.3. Statistical analyses included univariate and multivariate analyses. Differences between the two groups were evaluated using Student’s *t*-test. Multivariate analysis was conducted using SIMCA software (V16.0.2, Sartorius Stedim Data Analytics AB, Umeå, Sweden) for principal component analysis (PCA) and orthogonal partial least-squares discriminant analysis (OPLS-DA). In the OPLS-DA model, variables with VIP > 1 and *p*-value < 0.05 were considered significant differential metabolites. KEGG functional annotation and metabolic pathway enrichment analyses were performed using MetaboAnalyst 5.0 (https://www.metaboanalyst.ca/; accessed 2 November 2022).

### 2.6. Integrated Network-Pharmacology and Metabolomics Analysis

Interactions between differential metabolites and core targets were examined via an integrated analysis of network pharmacology and metabolomics using the “Joint Pathway Analysis” module of MetaboAnalyst 5.0. (https://www.metaboanalyst.ca/, accessed on 5 November 2024).

### 2.7. Molecular Docking

2D structures of the core compounds were downloaded from the PubChem database (https://pubchem.ncbi.nlm.nih.gov/, accessed on 8 November 2024) and converted to 3D conformers using ChemBioOffice Ultra 13.0.2-Chem3D. The resulting structures were energy-minimized. The protein structures corresponding to the core targets were retrieved from the PDB database (https://www.rcsb.org/, accessed on 8 November 2024) and processed (water and extraneous ligands were removed) using PyMOL 2.5.2. Docking grids were defined using AutoDockTools-1.5.7, ensuring that the grid box encompassed the entire protein. Semi-flexible molecular docking was performed using Vina (v. 1.2.5); the absolute value of the binding energy was used as the affinity metric, with higher values indicating stronger receptor–ligand interactions.

### 2.8. Statistical Analysis

All analyses were performed using SPSS software (version 26.0; IBM, Armonk, NY, USA). Results are presented as mean ± standard deviation (SD). Differences among groups were evaluated using one-way analysis of variance (ANOVA). Statistical significance was set at *p* < 0.05.

## 3. Results

### 3.1. Effect on Platelet Aggregation Rate in Rat Acute Blood Stasis Model

**Figure 1:**
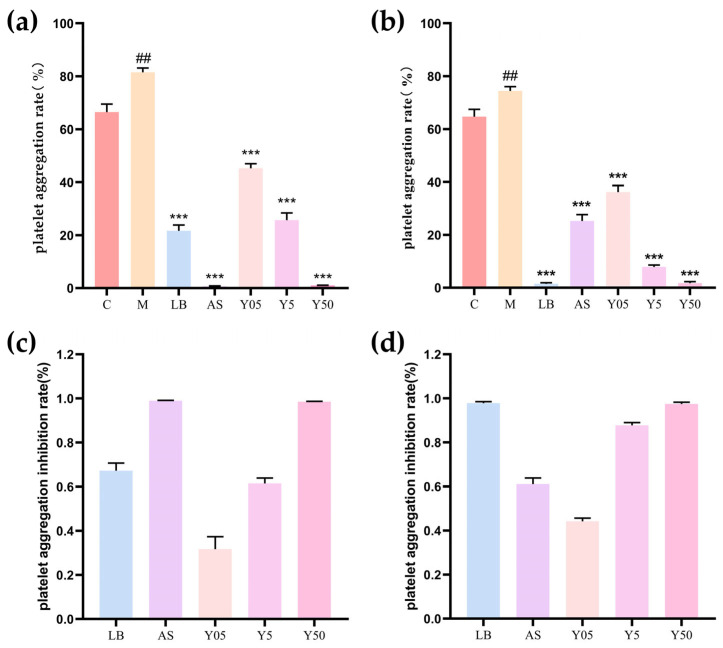
Platelet aggregation rates. (**a**) Platelet aggregation rates among groups induced by AA. (**b**) Platelet aggregation rates among groups induced by ADP. (**c**) Platelet aggregation inhibition rates induced by AA among all groups. (**d**) Platelet aggregation inhibition rates induced by ADP among all groups. ## indicates M group vs. C group, *p <* 0.01; *** indicates treatment group vs. M group, *p* < 0.001.

Arachidonic acid (AA) and adenosine diphosphate (ADP) are widely used as platelet aggregation inducers [24]. In this study, AA induced aggregation rates of 66.50% ± 2.96% in the C group, whereas those of the acute blood-stasis M group rose significantly to 81.57% ± 1.62% (vs. C group, *p* < 0.01). With ADP stimulation, the aggregation rates were 64.73% ± 2.81% and 74.43% ± 1.68%, respectively (vs. C group, *p* < 0.05), indicating that the acute blood-stasis model was successfully established (Figure 1). Further analysis revealed that, under AA stimulation, platelet aggregation in all drug-treated groups was markedly lower than that in the M group (*p* < 0.01), demonstrating a significant inhibitory effect on AA-induced aggregation. The strongest effects were observed in the AS and Y50 groups, whose aggregation rates decreased to 0.70% ± 0.10% and 1.00% ± 0.10%, respectively. The rates for the LB, Y05, and Y5 groups were 21.70% ± 2.08%, 45.30% ± 1.71% and 25.67% ± 2.74%. For corresponding inhibition ratios, AS (0.99 ± 0.01) and Y50 (0.98 ± 0.02) were the highest; among the CTS-dose groups, Y5 (0.61 ± 0.01) and Y50 exceeded LB (0.67 ± 0.03), while Y05 was the lowest (0.32 ± 0.06). With ADP stimulation, aggregation was likewise significantly reduced in all treated groups relative to the M group (*p* < 0.01), confirming pronounced inhibitory action against ADP-induced aggregation. The strongest effects were observed in the LB, Y5, and Y50 groups, whose aggregation values decreased to 1.43% ± 0.46%, 7.93% ± 0.61% and 1.70% ± 0.61%, respectively. The AS and Y05 groups exhibited weaker activity, yet their aggregation rates still decreased to 25.20% ± 2.46% and 36.17% ± 2.46%, respectively. Corresponding inhibition ratios ranked highest for LB (0.98 ± 0.01), Y50 (0.97 ± 0.01), and Y5 (0.88 ± 0.01), whereas Y05 (0.44 ± 0.01) remained lower than the former three but superior to AS (0.61 ± 0.03). Collectively, CTS significantly suppressed AA- and ADP-induced platelet aggregation in a dose-dependent manner.

### 3.2. Network Pharmacology Analysis

**Figure 2:**
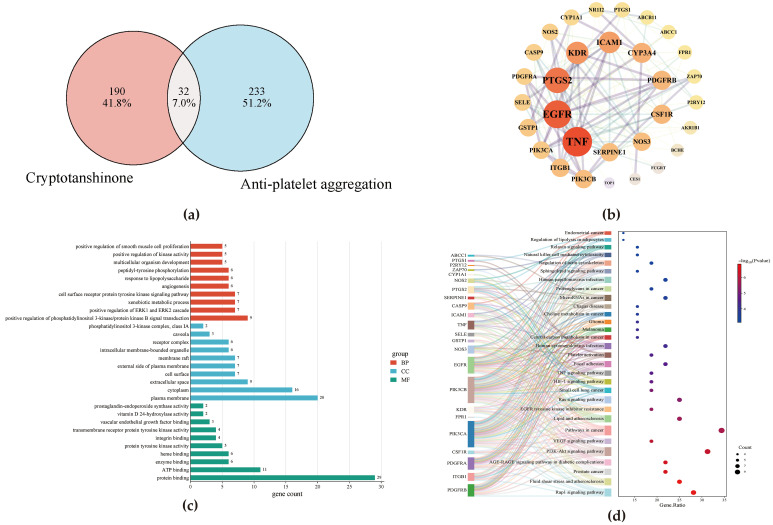
Network-pharmacology analysis. (**a**) Venn diagram of CTS and antiplatelet -aggregation targets. (**b**) PPI network of the intersecting targets. (**c**) GO enrichment analysis. (**d**) Sankey bubble plot of KEGG pathways.

Network pharmacology was employed to explore the potential mechanism by which CTS inhibits platelet aggregation in a rat model of acute blood stasis. In total, 222 drug-associated and 262 disease-related targets were retrieved, with 32 overlapping therapeutic targets (Figure 2a), which were used to construct a STRING-derived protein–protein interaction (PPI) network (Figure 2b). The top 15 nodes ranked by degree included TNF, EGFR, PTGS2, KDR, and ICAM1 (Table S1).

GO analysis returned 211 terms comprising 143 biological processes (BP), 27 cellular components (CC), and 41 molecular functions (MF) entries. The BP terms were enriched for receptor protein tyrosine kinase signaling pathway, xenobiotic metabolism, positive regulation of ERK1/ERK2, and phosphatidylinositol 3-kinase/protein kinase B (PI3K-Akt) signal transduction. The MF terms mainly included heme, enzyme, ATP, and protein binding. CC terms corresponding to the cell surface, the extracellular space, the cytoplasm, and the plasma membrane (Figure 2c). KEGG pathway analysis identified 93 signaling pathways, with the most significant ones including the Rap1 signaling pathway, fluid shear stress, atherosclerosis, PI3K-Akt signaling pathway, and cancer-related pathways (Figure 2d).

### 3.3. Untargeted Metabolomics

#### 3.3.1. Multivariate Statistical Analysis

**Figure 3:**
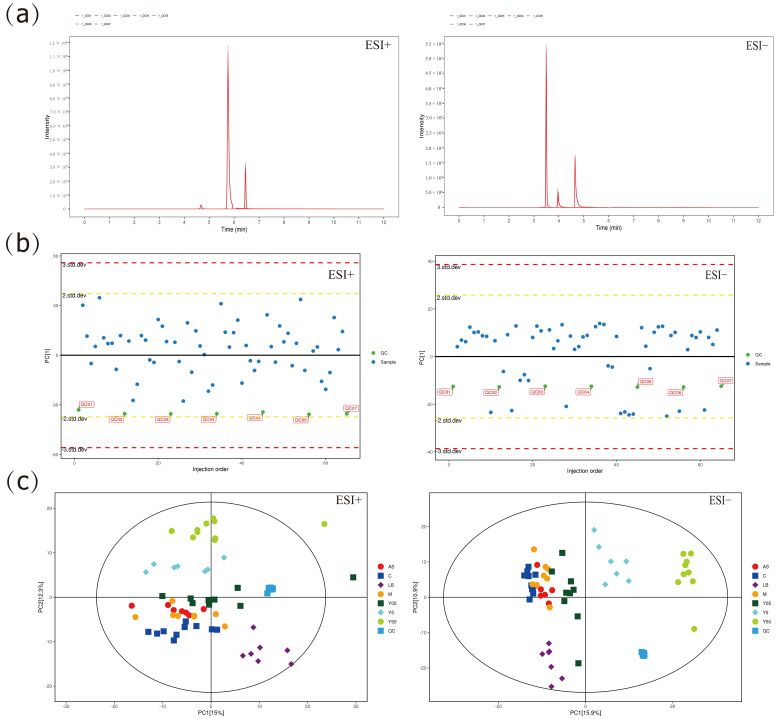
EIC and PCA score plots. (**a**) EIC of the internal standard in QC samples; (**b**) One-dimensional PCA-X distribution of QC samples; (**c**) PCA score plot of samples from all groups and QC replicates.

Metabolomic datasets are inherently high-dimensional and noise-laden; consequently, multivariate pattern recognition was employed to extract biologically relevant features. Inspection of the extracted-ion chromatograms (EICs) of the internal standards in all QC injections (Figure 3a) revealed negligible drift in retention time and response intensity, thereby confirming instrumental stability. Subsequent examination of the QC samples along the first principal component of a PCA-X model (Figure 3b) showed that every QC specimen resided within ±2 standard deviations of the centroid, validating reproducibility. The PCA score plot (Figure 3c) displays the metabolic profiles of the seven rat groups, together with the QC cohort. QC replicates clustered tightly, underscoring methodological robustness, whereas all experimental samples fell within the 95% confidence ellipse, with the seven groups clearly separated. Relative to the M group, the Y05, Y5, and Y50 groups converged sequentially, indicating a dose-dependent metabolic signature of CTS. These three treatment groups were spatially distinct from the positive control LB group cluster, suggesting the involvement of divergent metabolic pathways. Notably, the Y05 group was localized adjacent to the alternative positive control AS group, whereas the Y5 and Y50 groups deviated markedly from the AS group, indicating that low-dose CTS may partially recapitulate the metabolic actions of the AS group. These observations warrant confirmation by supervised OPLS-DA modeling and pathway enrichment analysis.

**Figure 4:**
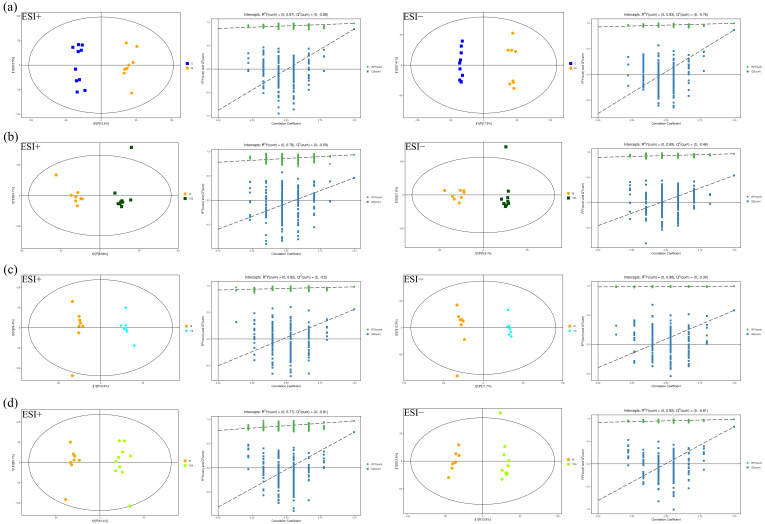
OPLS-DA score plots and permutation test scatter plots of rat serum metabolites in positive and negative ion modes. (**a**) C group vs. M group; (**b**) M group vs. Y05 group; (**c**) M group vs. Y5 group; (**d**) M group vs. Y50 group.

To further resolve inter-group metabolic differences and identify robust discriminant metabolites, orthogonal partial least squares discriminant analysis (OPLS-DA) was applied pairwise: C group vs. M group, M group vs. Y05 group, M group vs. Y5 group, and M group vs. Y50 group. Both ESI^+^ and ESI^−^ modes exhibit a clear separation along the first predictive component between C and M groups (Figure 4a), confirming the validity of the acute blood-stasis model. Likewise, the M group was distinctly segregated from the Y05, Y5, and Y50 groups (Figure 4b–d), with tight within-group clustering, indicating that CTS markedly remodeled the plasma metabolome of the model rats. The model robustness and predictive ability, evaluated using seven-fold cross-validation combined with 200-iteration permutation testing, showed R^2^Y values close to unity, corroborating the faithful representation of the observed data, Q^2^ intercepts of <0, with the majority of permuted Q^2^ values falling below the corresponding original points (Table S2), demonstrating the absence of overfitting and confirming model robustness. R^2^X denotes the variance in X explained by the model, R^2^Y quantifies the variance in class variable Y, and Q^2^ reflects the predictive accuracy.

#### 3.3.2. Screening and Identification of Differential Metabolites

**Figure 5:**
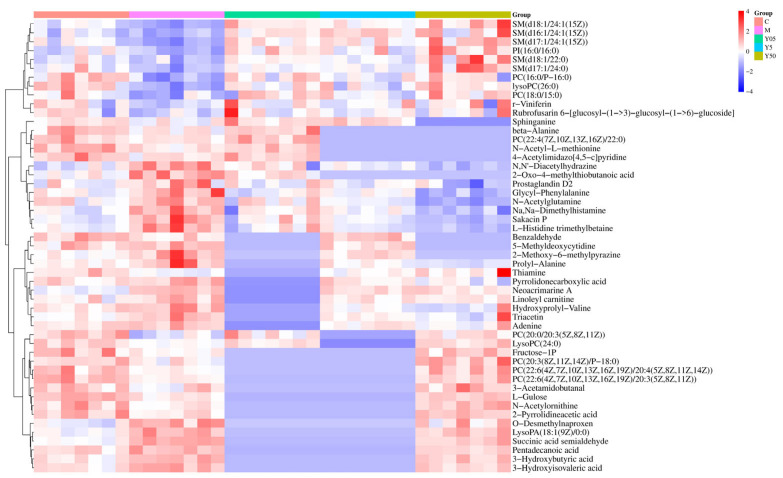
Heatmap of unsupervised clustering of 51 serum metabolic biomarkers.

Differential metabolites were selected by combining variable importance in projection (VIP) values from OPLS-DA models with two-tailed independent-samples t-tests; thresholds were set at VIP > 1 and *p <* 0.05. Volcano plots were constructed to visualize the overall expression patterns of the resulting features (Figures S1 and S2). Putative identifications were made by matching accurate mass and MS/MS fragmentation patterns against the Human Metabolome Database (HMDB). Compared to the C group, the M group showed 151 discriminant metabolites in the positive-ion mode (40 upregulated and 111 downregulated) and 83 in the negative-ion mode (42 upregulated and 38 downregulated). CTS administration significantly normalized 51 metabolites in M rats, including succinic acid semialdehyde, Neoacrimarine A, and 2-oxo-4-methylthiobutanoic acid (Table 1). Among these, the low-, medium-, and high-dose CTS groups normalized 27, 31, and 42 metabolites, respectively. A heat map of these 51 CTS-responsive metabolites (Figure 5) demonstrated dose-dependent restoration trends and distinct expression profiles of each experimental group. Functional categorization of these 51 metabolites revealed that 19 were directly associated with platelet activation pathways, while the remaining 32 were related to systemic metabolic stress, thereby delineating the targeted antiplatelet actions of CTS from its broader systemic effects.

#### 3.3.3. KEGG Pathway Analysis of Differential Metabolites

**Figure 6:**
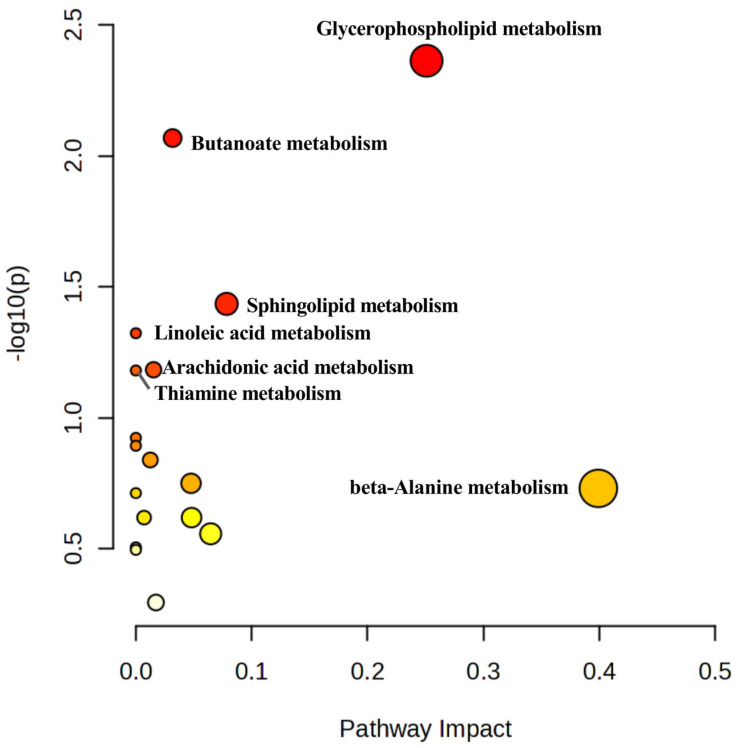
Enrichment of potential biomarker metabolic pathways.

To elucidate the biological relevance of the 51 differential metabolites, an integrated KEGG enrichment analysis, including over-representation and topology analyses, was performed using MetaboAnalyst 5.0. Pathways with impact > 0.1 and *p <* 0.05 were considered relevant, yielding seven key pathways (Table 2). These included glycerophospholipid metabolism, butanoate metabolism, sphingolipid metabolism, linoleic acid metabolism, arachidonic acid metabolism, thiamine metabolism, and β-alanine metabolism (Figure 6).

### 3.4. Integrated Analysis of Metabolomics and Network Pharmacology

**Figure 7:**
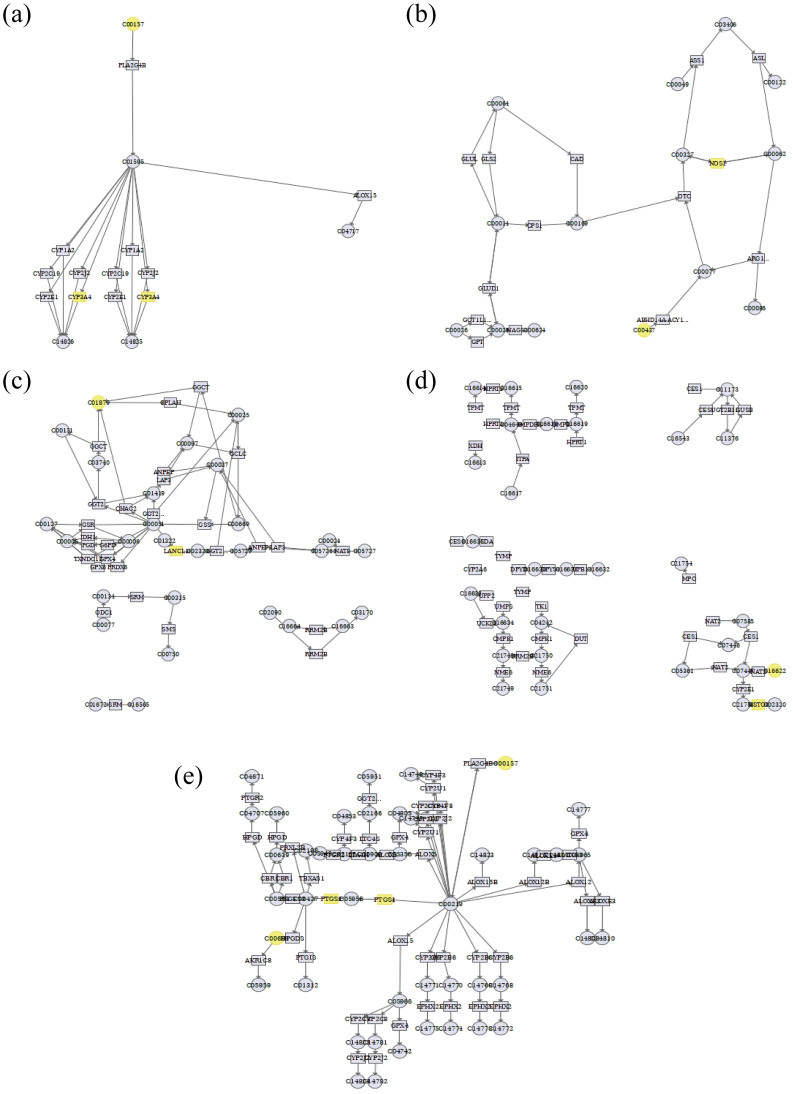
(**a**) Linoleic acid metabolism; (**b**) Arginine biosynthesis; (**c**) Glutathione metabolism; (**d**) Drug metabolism—other enzymes; (**e**) Arachidonic acid metabolism.

Differential metabolites and core targets identified by network pharmacology were uploaded to MetaboAnalyst 5.0 and subjected to Joint Pathway Analysis, which mapped both target sets onto KEGG pathways. Five common pathways emerged: linoleic acid metabolism, arginine biosynthesis, arachidonic acid metabolism, glutathione metabolism, and drug metabolism (Figure 7). CTS exerted its anti-platelet-aggregative effect primarily by modulating CYP3A4, NOS3, PTGS2, and GSTP1 within these five pathways (Table S3).

### 3.5. Analysis of Molecular Docking

**Figure 8:**
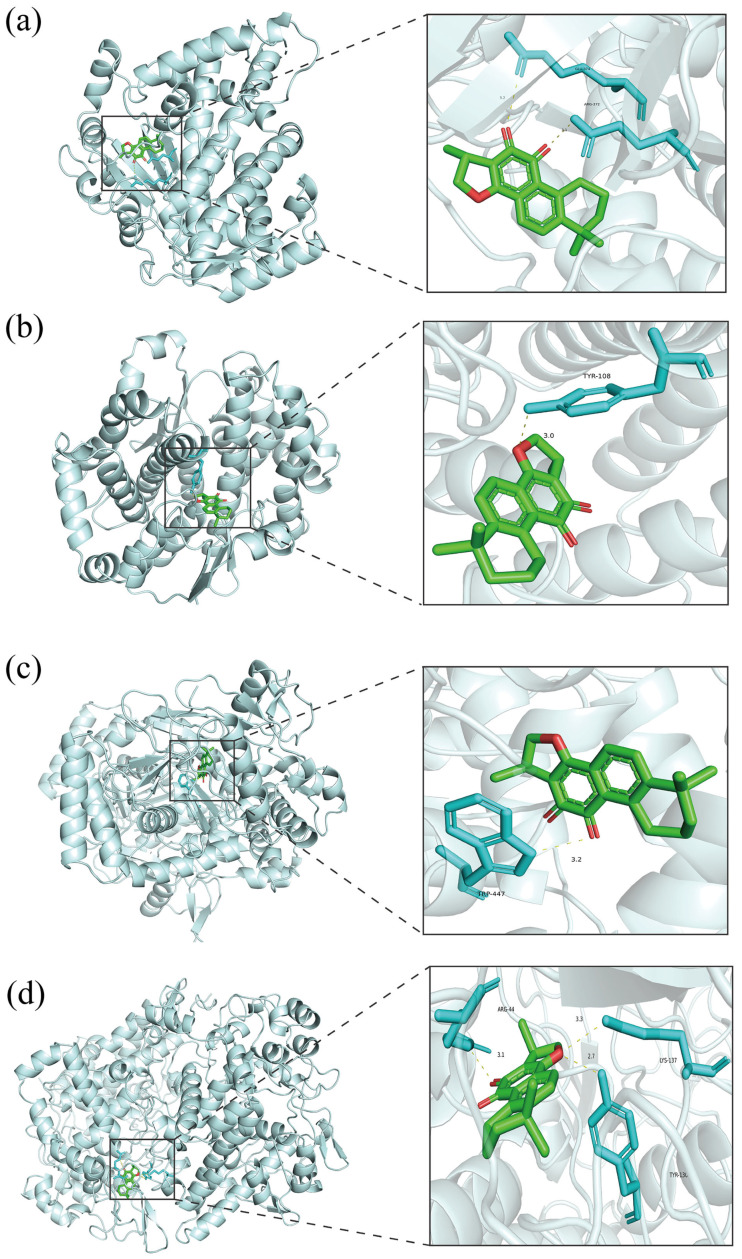
Molecular docking models. (**a**) CTS–CYP3A4; (**b**) CTS–GSTP1; (**c**) CTS–NOS3; (**d**) CTS–PTGS2. In the detailed view, green = ligand (cryptotanshinone), blue = receptor residues, dashed lines = hydrogen bonds.

CTS was docked against four core targets (CYP3A4, NOS3, PTGS2, and GSTP1) identified by integrated metabolomics and network pharmacology analysis using AutoDock Vina. The pose with the lowest binding energy was retained. Binding affinity was assessed using the calculated binding free energy, with more negative values indicating stronger ligand–receptor interactions. The resulting complexes were visualized in PyMOL 2.5.2 (Figure 8), where the dashed lines denote hydrogen bonds between the ligand and binding site residues. All four docking runs yielded binding energies lower than 9 kcal/mol. Table S4, demonstrating that CTS exhibits exceptionally high affinity for each of the identified targets.

## 4. Discussion

In this study, an adrenaline/ice-water-induced acute blood stasis model and platelet aggregation rates induced by AA and ADP were measured with turbidimetry. The acute blood stasis model effectively simulates an abnormal platelet aggregation state resulting from blood flow stagnation, reflecting the pathophysiological processes of platelet aggregation [25,26]. The results of this present study demonstrated that both aspirin and clopidogrel significantly inhibited AA- and ADP-induced platelet aggregation compared with the model group (*p <* 0.001). Specifically, aspirin exhibited a markedly superior inhibitory effect on AA-induced platelet aggregation compared to clopidogrel (*p <* 0.01), consistent with its known inhibitory mechanism: targeting the COX-1/TXA_2_ pathway [27]. In contrast, clopidogrel more effectively blocked ADP-induced aggregation (*p <* 0.01), aligning with its role as an irreversible P2Y_12_ receptor antagonist that blocks the ADP–P2Y_12_ signaling pathway [28]. However, CTS at medium and high doses significantly inhibited both AA- and ADP-induced platelet aggregation in a dose-dependent manner, suggesting its multi-pathway regulatory mechanism. Additionally, although early docking studies suggested that CTS might act as a Gi-coupled P2Y12 receptor antagonist, recent functional data have demonstrated that its anti-aggregatory effect is entirely independent of P2Y12 but is rather mediated by concurrent inhibition of the PI3K–AKT, MAPK, and STAT3 signaling networks [29,30].

Network pharmacology screening identified 15 core targets, providing clues for dissecting the multi-target characteristics of CTS. Untargeted metabolomics revealed 51 differential metabolites and seven key pathways, including glycerophospholipid, butanoate, and sphingolipid metabolism, which mirror the metabolic perturbations inherent in the acute blood stasis model. Integrative analysis cross-linked these two datasets and identified CYP3A4, NOS3, PTGS2, and GSTP1 as the four most relevant nodes governing the anti-platelet-aggregation effect. It must be emphasized that the four targets were not selected solely through computational prediction. Using MetaboAnalyst 5.0, we conducted a pathway-level integration of the 15 candidate proteins with the 51 CTS-modulated metabolites. The algorithm identifies metabolic pathways in which both (i) metabolites significantly reversed by CTS and (ii) the enzymes documented to catalyze these metabolites are concurrently enriched. Consequently, CYP3A4, NOS3, PTGS2, and GSTP1 surfaced from our omics dataset as the most proximal enzymatic nodes linking CTS intervention to the observed metabolic phenotype, thereby substantiating their candidacy as plausible mechanistic targets. Molecular-docking simulations showed strong binding energies between CTS and each of these targets (<−9 kcal/mol), indicating stable binding at the molecular level and implying potential inhibition or modulation of enzymatic activity. Such direct target engagement has been proposed to rebalance downstream metabolic circuits, ultimately converging on the observed anti-platelet-aggregation phenotype.

Linoleic acid, an essential ω-6 polyunsaturated fatty acid, is a structural component of membrane phospholipids and a key precursor of bioactive lipid mediators—including epoxy-octadecenoic acids (EpOMEs) and AA—which in turn modulate inflammation, vascular tone, and platelet aggregation [31,32]. In the present study, CTS restored the disrupted linoleic acid metabolism in rats with acute blood stasis. Molecular docking suggested a potential interaction with CYP3A4, a key enzyme in this pathway; however, whether CTS affects platelet function by regulating CYP3A4 and linoleic acid metabolism requires further verification. Based on this, it is hypothesized that CTS attenuates inflammation and improves vascular function, at least in part, by potentially suppressing the CYP3A4-mediated epoxy-ω-oxidation and modulating the formation of EpOMEs. This metabolic correction likely altered platelet membrane phospholipids, which can impair receptor localization and downstream signaling, thereby reducing platelet aggregation [33]. Therefore, CTS may jointly alleviate acute blood stasis by modulating bioactive lipid mediators and concomitantly remodeling the membrane phospholipid microenvironment.

Arginine is an immediate substrate for nitric oxide (NO) biosynthesis, and its bioavailability directly determines endothelial NO production [34]. NO synthase 3 (NOS3, also known as eNOS) catalyzes the conversion of L-arginine to NO, thereby maintaining vasodilation and suppressing platelet activation and aggregation [35]. Impaired arginine metabolism reduces NO generation and consequently favors platelet hyperreactivity [36]. Molecular docking analyses suggested that CTS interferes with NOS3, thereby attenuating L-arginine-to-NO conversion and ultimately inhibiting platelet aggregation.

AA metabolism constitutes a canonical signaling cascade in platelet activation, and the balance between its downstream products, thromboxane A_2_ (TXA_2_) and prostacyclin (PGI_2_), largely determines the extent of platelet aggregation [37]. However, the TXA_2_/PGI_2_ equilibrium is not governed solely by platelet COX-1 [38]. Under acute blood-stasis conditions, inflammatory cytokines markedly induce COX-2 expression in vascular endothelial and infiltrating immune cells, expanding the local prostaglandin H_2_ (PGH_2_) pool [39]. Excess PGH_2_ is preferentially channeled by thromboxane-A synthase (TXAS) toward TXA_2_ and, less efficiently converted to PGI_2_ by prostacyclin synthase (PGIS), thereby amplifying the pro-thrombotic milieu [40]. The present data demonstrate that CTS normalizes AA metabolite profiles in rats with experimentally induced acute blood stasis, integrative “metabolite-target” mapping identified PTGS2 (COX-2) as a key target, and molecular docking revealed a robust binding affinity of −10.5 kcal mol^−1^ between CTS and COX-2. These findings suggest that CTS attenuates platelet aggregation partly by inhibiting COX-2 activity, diminishing endothelial/inflammatory-cell-derived PGH_2_ formation, and consequently blunting paracrine TXA_2_ signaling.

Glutathione metabolism maintains cellular redox homeostasis, with the depletion of its metabolite, glutathione (GSH), precipitating reactive oxygen species (ROS) accumulation, endothelial injury, and platelet activation [41]. During acute blood stasis, severe oxidative stress consumes large amounts of GSH and markedly elevates intracellular ROS [42]. The ensuing GSH decline activates platelet NADPH oxidase (NOX) and phospholipase A_2_, thereby enhancing the generation of pro-aggregatory mediators, including TXA_2_ and 8-isoprostane. In the vascular endothelium, GSH modulates eNOS activity to augment NO production, thereby establishing a dual defense against platelet aggregation [43]. In the present study, GSTP1 was identified as a key target, and molecular docking suggests that CTS could potentiate GSTP1-mediated GSH regeneration, thereby reducing ROS-driven platelet activation and contributing an antioxidant component to its antiplatelet effect.

In addition to xenobiotic detoxification, drug-metabolizing enzymes modulate platelet function by processing endogenous substrates such as AA. Integrative profiling highlighted GSTP1 in the “drug metabolism—other enzymes” category [44]. Docking simulations revealed a robust binding between CTS and GSTP1, implying that CTS regulates GSTP1-driven phase II metabolism and glutathione conjugation. This interaction can simultaneously alter detoxification efficiency and redox homeostasis, thereby indirectly restraining platelet activation and aggregation.

Although the integrated network pharmacology and metabolomics in this study revealed multiple CTS targets, functional validations are needed to confirm their contribution to platelet inhibition. Future work should focus on directly testing this multi-target hypothesis through in vitro and in vivo experiments designed to measure the functional impact of CTS on the activity and expression of CYP3A4, NOS3, PTGS2, and GSTP1.

## 5. Conclusions

By integrating network pharmacology with untargeted metabolomics, we identified that CTS simultaneously targets CYP3A4, NOS3, PTGS2, and GSTP1, thereby reprogramming arachidonic acid and glutathione metabolism and suppressing platelet aggregation at multiple nodes. This mechanism explains its therapeutic efficacy in the acute blood stasis rat model and provides quantifiable metabolic biomarkers for dose optimization. Collectively, CTS represents a promising multi-target antiplatelet agent; however, the target-specific functions require experimental validation.

## Figures and Tables

**Table 1:** Potential platelet aggregation-related differential metabolites significantly modulated by CTS.

NO.	RT (s)	*m*/*z*	HMDB ID	Metabolite	Classification	Lon Mode	Trend			
							C vs. M	M vs. Y05	M vs. Y5	M vs. Y50
1	217.5330	101.0241	HMDB0001259	Succinic acid semialdehyde	B	NRG	↑	-	-	↓
2	431.1600	682.2992	HMDB0040384	Neoacrimarine A	B	POS	↑	-	↓	↓
3	216.8970	435.2512	HMDB0007855	LysoPA(18:1(9Z)/0:0)	A	NRG	↑	-	-	↓
4	78.9896	147.0117	HMDB0001553	2-Oxo-4-methylthiobutanoic acid	B	NRG	↑	↓	-	-
5	160.3015	840.6418	HMDB0008277	PC(20:0/20:3(5Z,8Z,11Z))	A	POS	↓	↑	-	↑
6	166.0875	718.5734	HMDB0007994	PC(16:0/P-16:0)	A	POS	↓	↑	↑	↑
7	407.2230	217.1174	HMDB0038239	Sakacin P	B	POS	↑	↓	↓	↓
8	286.5010	103.0398	HMDB0000357	3-Hydroxybutyric acid	B	NRG	↑	-	-	↓
9	197.6575	813.6816	HMDB0012107	SM(d18:1/24:1(15Z))	A	POS	↓	↑	↑	↑
10	347.4185	115.0512	HMDB0060496	N,N′-Diacetylhydrazine	B	NRG	↑	↓	↓	↓
11	248.1410	215.0725	HMDB0013989	O-Desmethylnaproxen	B	NRG	↑	-	-	↓
12	308.3890	203.0517	HMDB0012326	L-Gulose	B	POS	↓	-	-	↑
13	291.6910	198.1228	HMDB0029422	L-Histidine trimethylbetaine	B	POS	↑	↓	↓	↓
14	155.2270	856.5802	HMDB0008737	PC(22:6(4Z,7Z,10Z,13Z,16Z,19Z)/20:3(5Z,8Z,11Z))	A	POS	↓	-	-	↑
15	293.1655	140.1176	HMDB0033438	Na,Na-Dimethylhistamine	B	POS	↑	↓	↓	↓
16	355.6875	265.1105	HMDB0000235	Thiamine	B	POS	↓	-	↑	↑
17	206.5500	608.4628	HMDB0010405	LysoPC(24:0)	A	POS	↓	↑	-	↑
18	198.0780	799.6664	HMDB0011696	SM(d17:1/24:1(15Z))	A	POS	↓	↑	↑	↑
19	205.1945	636.4930	HMDB0029205	lysoPC(26:0)	A	POS	↓	↑	↑	↑
20	379.5950	219.0842	HMDB0029592	Triacetin	B	POS	↑	-	↓	↓
21	214.6055	117.0554	HMDB0000754	3-Hydroxyisovaleric acid	B	NRG	↑	-	-	↓
22	153.9160	854.5663	HMDB0008739	PC(22:6(4Z,7Z,10Z,13Z,16Z,19Z)/20:4(5Z,8Z,11Z,14Z))	A	POS	↓	-	-	↑
23	168.0830	748.5792	HMDB0008033	PC(18:0/15:0)	A	POS	↓	↑	↑	↑
24	221.2975	809.5157	HMDB0009778	PI(16:0/16:0)	A	NRG	↓	↑	↑	↑
25	290.7780	223.1066	HMDB0028848	Glycyl-Phenylalanine	B	POS	↑	↓	↓	↓
26	373.0340	175.1067	HMDB0003357	N-Acetylornithine	B	POS	↓	-	-	↑
27	151.9330	125.0703	HMDB0040143	2-Methoxy-6-methylpyrazine	B	POS	↑	-	↓	-
28	198.3560	801.6802	HMDB0011695	SM(d17:1/24:0)	A	POS	↓	↑	↑	↑
29	232.7680	107.0488	HMDB0006115	Benzaldehyde	B	POS	↓	-	↑	-
30	380.6705	88.0403	HMDB0000056	beta-Alanine	B	NRG	↓	↑	-	-
31	101.5005	130.0856	HMDB0029444	2-Pyrrolidineacetic acid	B	POS	↓	-	-	↑
32	313.2795	179.0555	HMDB0062538	Fructose-1P	B	NRG	↓	-	-	↑
33	169.9790	136.0612	HMDB0000034	Adenine	B	POS	↑	-	↓	↓
34	394.3850	231.1329	HMDB0028876	Hydroxyprolyl-Valine	B	POS	↑	-	↓	↓
35	52.7136	130.0856	HMDB0059649	3-Acetamidobutanal	B	POS	↓	-	-	↑
36	404.3080	189.0861	HMDB0006029	N-Acetylglutamine	B	POS	↑	↓	↓	↓
37	197.0020	424.3403	HMDB0006469	Linoleyl carnitine	A	POS	↑	-	↓	↓
38	28.4327	907.2543	HMDB0041291	r-Viniferin	B	POS	↓	↑	↑	↑
39	45.2212	351.2199	HMDB0001403	Prostaglandin D2	A	NRG	↑	↓	↓	↓
40	45.7435	241.2167	HMDB0000826	Pentadecanoic acid	B	NRG	↑	-	-	↓
41	150.6600	162.0654	HMDB0034888	4-Acetylimidazo[4,5-c]pyridine	B	POS	↓	↑	-	-
42	211.7070	242.1124	HMDB0002224	5-Methyldeoxycytidine	B	POS	↑	-	↓	-
43	215.8790	190.0536	HMDB0011745	N-Acetyl-L-methionine	B	NRG	↓	↑	-	-
44	42.3529	302.3035	HMDB0000269	Sphinganine	A	POS	↓	↑	↑	-
45	316.9175	187.1067	HMDB0029010	Prolyl-Alanine	B	POS	↑	-	↓	↓
46	272.4340	130.0492	HMDB0000805	Pyrrolidonecarboxylic acid	B	POS	↑	-	↓	↓
47	28.5982	759.2177	HMDB0034569	Rubrofusarin 6-[glucosyl-(1->3)-glucosyl-(1->6)-glucoside]	B	POS	↓	↑	↑	↑
48	156.3890	796.6173	HMDB0008423	PC(20:3(8Z,11Z,14Z)/P-18:0)	A	POS	↓	-	-	↑
49	198.7930	785.6506	HMDB0011694	SM(d16:1/24:1(15Z))	A	POS	↓	↑	↑	↑
50	199.5415	787.6648	HMDB0012103	SM(d18:1/22:0)	A	POS	↓	↑	↑	↑
51	153.3870	894.6874	HMDB0008643	PC(22:4(7Z,10Z,13Z,16Z)/22:0)	A	POS	↓	↑	-	-

↑ indicates significant up-regulation, ↓ indicates significant down-regulation, and - indicates not detected. A represents platelet-directly related metabolites; B represents non-specific metabolites.

**Table 2:** Metabolic pathways enriched by potential biomarkers and related information.

Pathway Name	Total	Hits	*p*	FDR	Impact
Glycerophospholipid metabolism	36	3	0.00434	0.34238	0.25096
Butanoate metabolism	15	2	0.00856	0.34238	0.03175
Sphingolipid metabolism	32	2	0.036766	0.88018	0.07838
Linoleic acid metabolism	5	1	0.04758	0.88018	0
Arachidonic acid metabolism	44	2	0.065589	0.88018	0.01528
Thiamine metabolism	7	1	0.066014	0.88018	0
beta-Alanine metabolism	21	1	0.18602	1	0.39925

## Data Availability

The data presented in this study are available on request from the corresponding author. The data are not publicly available due to privacy or ethical restrictions.

## Supplementary Materials

The following supporting information can be downloaded at: https://www.mdpi.com/article/10.3390/cimb47110953/s1.

## Abbreviations

The following abbreviations are used in this manuscript:

CTS	Cryptotanshinone
AA	Arachidonic acid
ADP	Adenosine diphosphate
LB	Clopidogrel (positive control group)
PPI	Protein-protein interaction
GO	Gene Ontology
KEGG	Kyoto Encyclopedia of Genes and Genomes
BP	Biological process
CC	Cellular component
MF	Molecular function
QC	Quality control
PCA	Principal component analysis
OPLS-DA	Orthogonal partial least squares discriminant analysis
HMDB	Human Metabolome Database
EpOMEs	Epoxy-octadecenoic acids
NOX	NADPH oxidase
