# Supplementary material for: Integrating Network Pharmacology and Metabolomics to Elucidate the Mechanism of Cryptotanshinone Against Platelet Aggregation
Source: Curr Issues Mol Biol. 2025 Nov 17;47(11):953. doi: 10.3390/cimb47110953 (PMC12650884; doi:10.3390/cimb47110953)
Supplement: Supplementary file 1 [file cimb-47-00953-s001.zip › cimb-3948564-supplementary.pdf]

Table S1. Top 15 Hub Targets in the PPI Network by Degree Value.

NO.	target	Degree
1	TNF	24
2	EGFR	22
3	PTGS2	19
4	KDR	15
5	ICAM1	14
6	CYP3A4	12
7	CSF1R	11
8	PDGFRB	11
9	NOS3	10
10	PIK3CB	10
11	SERPINE1	10
12	ITGB1	10
13	GSTP1	9
14	PIK3CA	9
15	CASP9	8

Table S2. OPLS-DA Models Information.

Title	R2X(cum)	R2Y(cum)	Q2(cum)	lon mode
M-C	0.253	0.996	0.864	NEG
Y05-M	0.316	0.966	0.535	NEG
Y5-M	0.239	0.993	0.581	NEG
Y50-M	0.274	0.986	0.823	NEG
M-C	0.35	0.978	0.856	POS
Y05-M	0.428	0.932	0.459	POS
Y5-M	0.351	0.978	0.554	POS
Y50-M	0.395	0.969	0.738	POS

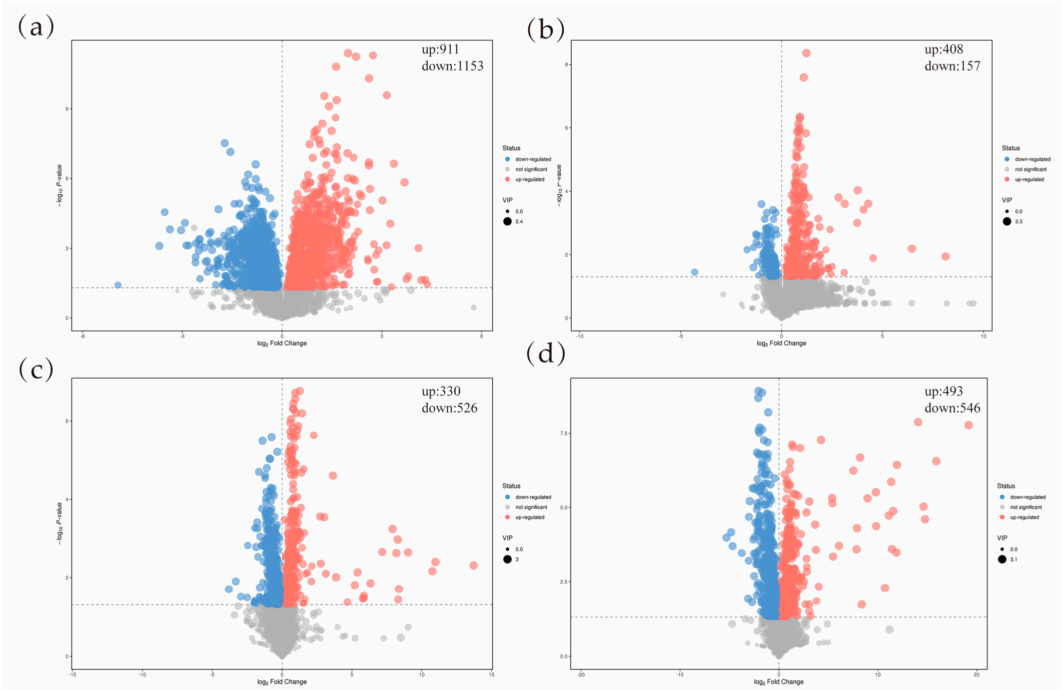

Figure S1. Volcano plots of metabolites (positive ion mode). (a) C group vs. M group; (b) M group vs. Y05 group; (c) M group vs. Y5 group; (d) M group vs. Y50 group.

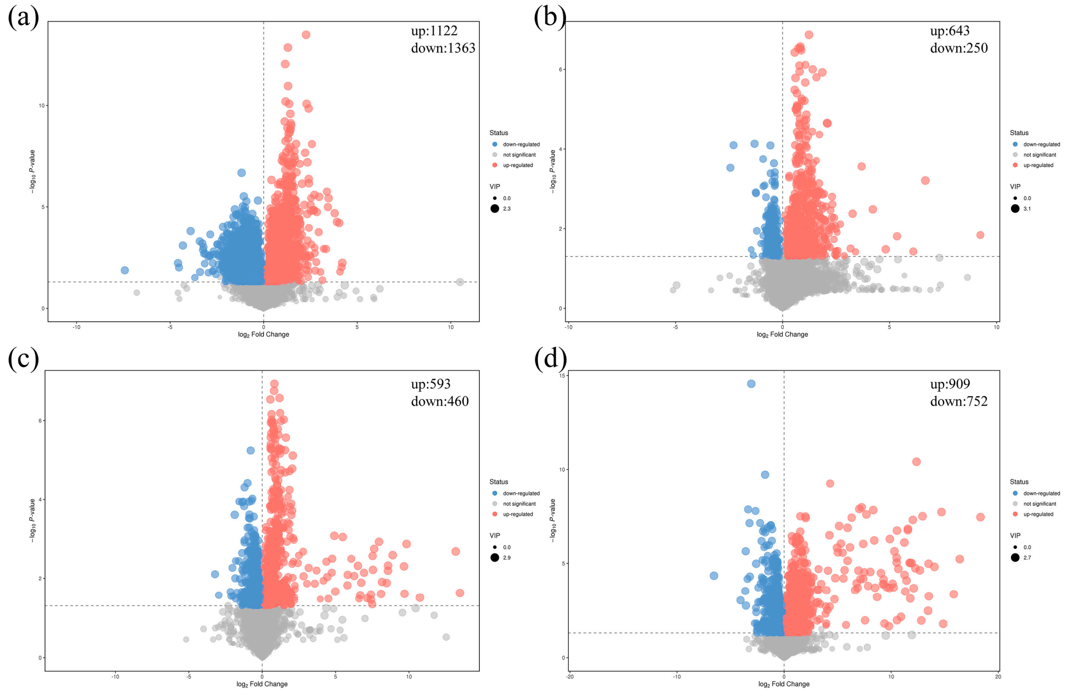

Figure S2. Volcano plots of metabolites (negative ion mode). (a) C group vs. M group; (b) M group vs. Y05 group; (c) M group vs. Y5 group; (d) M group vs. Y50 group.

Table S3. Integrated Pathway Analysis: Differentially Accumulated Metabolites and Their Associated Targets.

Pathway	metabolite	Target
Linoleic acid metabolism	Phosphatidylcholine	CYP3A4
Arginine biosynthesis	N2-Acetylornithine	NOS3
Arachidonic acid metabolism	Phosphatidylcholine，<br>Prostaglandin D2	PTGS2
Glutathione metabolism	Pyroglutamic acid	GSTP1
Drug metabolism - other enzymes	N,N'-Diacetylhydrazine	GSTP1

Table S4. Molecular Docking Results of Cryptotanshinone with Core Targets.

Target	PDB ID	specific forms	Binding Affinity （kcal/mol）
CYP3A4	1TQN	Chains:1<br>Resolution: 2.05 Å<br>Method: X-RAY DIFFRACTION<br>Organism(s): Homo sapiens<br>Mutation(s): No	-10.8
NOS3	1M9R	Chains:A,B<br>Resolution: 1.96 Å<br>Method: X-RAY DIFFRACTION<br>Organism(s): Homo sapiens<br>Mutation(s): No	-9.5
PTGS2	5F19	Chains:A,B<br>Resolution: 2.04 Å<br>Method: X-RAY DIFFRACTION<br>Organism(s): Homo sapiens<br>Mutation(s): No	-10.5
GSTP1	2GSS	Chains:A,B<br>Resolution: 1.90 Å<br>Method: X-RAY DIFFRACTION<br>Organism(s): Homo sapiens<br>Mutation(s): No	-9.7

All proteins undergo preprocessing of water removal and hydrogen addition
